# Identifying health-related quality of life cut-off scores that indicate the need for supportive care in young adults with cancer

**DOI:** 10.1007/s11136-022-03139-6

**Published:** 2022-04-27

**Authors:** Emma Lidington, Johannes M. Giesinger, Silvie H. M. Janssen, Suzanne Tang, Sam Beardsworth, Anne-Sophie Darlington, Naureen Starling, Zoltan Szucs, Michael Gonzalez, Anand Sharma, Bhawna Sirohi, Winette T. A. van der Graaf, Olga Husson

**Affiliations:** 1 The Royal Marsden NHS Foundation Trust, London, UK; 2 University Hospital of Psychiatry II, Medical University of Innsbruck, Innsbruck, Austria; 3 Department of Medical Oncology, Netherlands Cancer Institute – Antoni van Leeuwenhoek, Amsterdam, The Netherlands; 4 Department of Psychosocial Research and Epidemiology, Netherlands Cancer Institute – Antoni van Leeuwenhoek, Amsterdam, The Netherlands; 5 Royal Free London NHS Foundation Trust, London, UK; 6 London, UK; 7 Health Sciences, University of Southampton, Southampton, UK; 8 Division of Clinical Studies, Institute of Cancer Research, Sutton, UK; 9 Ipswich Hospital, East Suffolk and North Essex NHS Foundation Trust, Ipswich, UK; 10 Charing Cross Hospital, Imperial College Healthcare NHS Trust, London, UK; 11 Mount Vernon Hospital, The Hillingdon Hospitals NHS Foundation Trust, Northwood, UK; 12 St Bartholomew’s Hospital, Barts Health NHS Trust, London, UK; 13 Apollo Proton Cancer Centre, Chennai, India

**Keywords:** Quality of life, Patient-reported outcomes, Routine care, Supportive care needs, Oncology, Young adult

## Abstract

**Purpose:**

Using patient-reported outcomes in routine cancer care may improve health outcomes. However, a lack of information about which scores are problematic in specific populations can impede use. To facilitate interpretation of the European Organisation for Research and Treatment of Cancer Core Questionnaire (EORTC QLQ-C30), we identified cut-off scores that indicate need for support by comparing each scale to relevant items from the Supportive Care Needs Survey (SCNS-LF59) in a young adult (YA) population.

**Methods:**

We conducted a cross-sectional survey amongst YAs with cancer ages 25–39 at diagnosis. Participants completed the EORTC QLQ-C30 and SCNS-LF59. Patient, clinician and research experts matched supportive care needs from the SCNS-LF59 to quality of life domains of the EORTC QLQ-C30. We evaluated the EORTC QLQ-C30 domain score’s ability to detect patients with need using receiver operator characteristic (ROC) analysis, calculating the area under the ROC curve and sensitivity and specificity for selected cut-offs. Cut-offs were chosen by maximising Youden’s J statistic and ensuring sensitivity passed 0.70. Sensitivity analyses were conducted to examine the variability of the cut-off scores by treatment status.

**Results:**

Three hundred and forty-seven YAs took part in the survey. Six experts matched SCNS-LF59 items to ten EORTC QLQ-C30 domains. The AUC ranged from 0.78 to 0.87. Cut-offs selected ranged from 8 (Nausea and Vomiting and Pain) to 97 (Physical Functioning). All had adequate sensitivity (above 0.70) except the Financial Difficulties scale (0.64). Specificity ranged from 0.61 to 0.88. Four of the cut-off scores differed by treatment status.

**Conclusion:**

Cut-offs with adequate sensitivity were calculated for nine EORTC QLQ-C30 scales for use with YAs with cancer. Cut-offs are key to interpretability and use of the EORTC QLQ-C30 in routine care to identify patients with supportive care need.

## Plain English summary

Asking patients with cancer to complete questionnaires about their health and using the information in routine care to support clinical decision-making has shown a number of health and care benefits. However, for one commonly used cancer-related questionnaire (EORTC QLQ-C30), it’s unknown which scores indicate there is a problem, and which scores do not and whether this is unique to specific groups of people. Here we aim to identify cut-off scores for each symptom and function measured by the EORTC QLQ-C30 for young adults with cancer ages 25–39. This score indicates that any respondents scoring worse than the cut-off needs support from the healthcare team. Three hundred and forty-seven patients took part. By comparing the symptom and function scores to questions from another questionnaire which measures need for support, we identified cut-offs for nine of the fifteen scores. For five of the functions measured (Global Quality of Life, Physical Functioning, Emotional Functioning, Role Functioning and Social Functioning) where higher scores indicate better functioning, results below the cut-offs identified indicate the need for support. For four of the symptoms measured (Fatigue, Nausea and Vomiting, Pain and Insomnia) where higher scores indicate more problems, results above the cut-offs identified indicate supportive care need. These cut-off scores can facilitate the use of the EORTC QLQ-C30 in routine cancer care to identify supportive care need amongst young adults.

## Introduction

Interest in using patient-reported outcomes (PROs) in routine cancer care has increased dramatically in recent years [1]. PROs are direct reports from patients about symptoms, function or well-being with respect to a condition or treatment without interpretation by a clinician or anyone else [2]. Evidence suggests that using PROs in routine care may improve patient-clinician communication, quality of life, symptom burden, patient satisfaction and even survival [3–7]. In these cases, PROs are essentially used as screening tools to help identify problems for further discussion with a clinician that might otherwise go unaddressed.

Despite the potential benefit, using PROs in routine care can be challenging due to difficulty interpreting scores which are usually presented as a range of numerical values (i.e. 0–100) [8]. In cancer, most PROs have been designed for use in research where analysis can focus on group comparisons or change over time. However, in routine care, clinicians need to interpret scores at an individual level at a single time point. This requires an understanding of which scores are considered moderate or severe and require clinical attention. We can aid this interpretation by defining a ‘cut-off’ score, the threshold above or below which the scores are problematic.

The European Organisation for Research and Treatment of Cancer Quality of Life Core Questionnaire (EORTC QLQ-C30) is one of the PROs most commonly used in routine care [3]. However, research defining cut-offs for the questionnaire domains is limited.

Giesinger et al. identified cut-offs for functional and symptom domains by comparing patient scale scores on the EORTC QLQ-C30 to their responses on three external questions designed to reflect clinical importance (‘Has your symptom/problem limited your daily life?’, ‘Have you needed any help or care because of your symptom/problem?’ and ‘Has your symptom/problem caused you or your family/partner to worry?’) [9, 10]. Clinical importance was conceptualised as the need for clinical interaction, incorporating the presence of symptoms or problems that are limiting, the need for help or care and worries about the issues.

Snyder et al. instead took a more focused approach to screen for supportive care needs by comparing EORTC QLQ-C30 domain scores to selected items from a validated measure of supportive care need amongst cancer patients [11–13]. However, the short form of the supportive care needs survey (SCNS) used had a limited number of items that were conceptually similar to the EORTC QLQ-C30 domains. This meant the authors could only calculate adequate cut-offs for six of the 15 scales. Additionally, the sample included mostly older adults (mean age 61), which could limit generalisability. Cut-offs may be different amongst younger people as patients may have higher expectations for function and symptoms. For example, the optimal cut-off for social functioning in the study by Giesinger et al. for patients younger than 60 was 16 points lower than the optimal cut-off for older patients [14]. The lack of inclusion of young adults (YAs) in these studies likely obscures important differences in the identification of problematic scores.

Stronger evidence for cut-offs indicating the need for support for each domain will facilitate its use in standard cancer care as a screening tool for supportive care. We aim here to expand on the analysis by Snyder et al. to identify additional cut-offs on EORTC QLQ-C30 scale scores in a population of young adults (YAs) diagnosed with cancer between the ages of 25 and 39. We will replicate the analysis using the SCNS Long Form (SCNS-LF59) to utilise questions not found in the short form that may be relevant to additional EORTC QLQ-C30 scale scores (i.e. Nausea and Vomiting) and to the younger population (i.e. ‘fear of losing independence’).

## Materials and methods

### Study population and procedures

We conducted a multi-centre, cross-sectional survey where clinical teams invited potential participants by post between May 2018 and March 2019. Patients were eligible if they received a first primary cancer diagnosis of any type between the ages of 25 and 39 at one of the six participating centres in Southeast England between May 2013 and May 2018. Patients were excluded if the treating clinician determined they had severe cognitive disability or were physically too unwell (i.e. nearing end of life). Patients could complete the questionnaires by paper or online using PROFILES, a web-based system for the collection of patient-reported outcomes in cancer research [15]. To use the data for exploratory analyses, no formal sample size was calculated a priori but we aimed to enrol 350 patients based on number of eligible YAs at participating centres and expected 25% response [16].

### Measures

Participants reported demographic and clinical information including current age, age at diagnosis, gender, ethnicity, education, work status, cancer diagnosis, treatments, treatment status and treatment intent.

#### Supportive care needs

Participants completed the SCNS-LF59 as a measure of supportive care need [17]. This instrument includes 59 items which comprise five domains (psychological, health system and information, physical and daily living, patient care and support, and sexuality needs) and four single items (talking to other people, changes in others’ attitudes or behaviour towards you, financial concerns, transport). Response categories range from one to five and correspond to not applicable, satisfied, low need, moderate need and high need. Domain scores, calculated only if at least half the items are complete, are the average of all items in each domain.

#### Quality of life

Participants completed the EORTC QLQ-C30 to measure cancer-related quality of life [18]. The EORTC QLQ-C30 is a 30-item instrument with 15 scales in total: five functional scales (physical, emotional, cognitive, role, and social functioning), nine symptom scales (fatigue, pain, nausea and vomiting, dyspnoea, insomnia, appetite loss, constipation, diarrhoea and financial difficulties) and a global quality of life score. Scales are scored according to the manual if at least half the items are complete [19]. Scores range from 0 to 100. Higher scores on functional scales indicate better function, higher scores on the global quality of life scale indicates better quality of life, and higher scores on symptom scales indicate worse symptom burden.

### Anchor selection

To determine the cut-offs, each EORTC QLQ-C30 scale needed to be compared to a conceptually similar single item, composite item or domain from the SCNS-LF59, referred to as an anchor. To ensure selected anchors were strong conceptual matches to the EORTC QLQ-C30 scales, we involved six experts in a multi-round rating process.

Potential anchors previously suggested by Snyder et al. for each EORTC QLQ-C30 scale formed the starting point. New items found in the SCNS-LF59 compared to the short form were added as potential anchors to relevant scales based on conceptual similarity by the lead author (EL). Potential anchors were then reduced to a single anchor per scale in an iterative process with patient, clinician and researcher experts.

Experts were identified and approached based on previous collaborations on YA oncology and quality of life projects. Experts were provided with an overview of the study’s aims, methods, samples of the EORTC QLQ-C30 and SCNS-LF59 and a Microsoft Excel template for rating the anchors. The template first showed which items belonged to each scale in the EORTC QLQ-C30 and SCNS-LF59 to familiarise the experts with the questionnaires at item and domain-level. The template then had a page, which showed all the potential anchors for each EORTC QLQ-C30 scale. Instructions were discussed by phone where possible.

In the first round, experts were asked to independently rate each potential anchor in order of best conceptual fit, excluding items they thought did not fit entirely. If an expert thought the combination of every item in a domain matched the scale well, the domain could be selected and all single items excluded. Experts were also encouraged to add further potential anchors from the SCNS-LF59 if identified. The ratings were then returned and combined into a single document with each rating labelled only with a coded ID number for review by the lead author (EL).

Where there was majority agreement (4/6) that an anchor should be excluded, it was recorded as ‘excluded’, highlighted in red and grouped together. Where there was majority agreement that an anchor should be included, it was recorded as ‘included’, highlighted in green and grouped together. If there was agreement that a domain should be used as an anchor, it was included and all single items comprising that domain were excluded and treated as above. Newly suggested potential anchors were added in red text and grouped together with items that did not reach agreement for inclusion or exclusion. The spreadsheet with all the results was then presented back to the experts for a second round of ratings where they were asked specifically to rate the newly proposed items and those that had not reach agreement in the first round. This process continued until majority agreement was reached for each anchor.

### Statistical analysis

Descriptive statistics were calculated for demographic and clinical variables. Mean and standard deviation are presented for continuous variables. Frequency and percentages are presented for categorical variables. Patients with incomplete data were excluded.

First we dichotomised each anchor from the SCNS-LF59 selected by the experts. Scores > 2 indicate need whilst scores ≤ 2 indicate no need. Where more than one single item was chosen as an anchor, we calculated and dichotomised the mean score of the single items.

Cut-offs were evaluated using receiver operating characteristic (ROC) analysis, which allows us to evaluate the performance of a numerical test to classify subjects on a binary outcome [20]. The area under the ROC curve (AUC) indicates how well the numerical test can discriminate between the two binary outcomes levels [21]. Sensitivity (true positive rate) and specificity (true negative rate) can then be calculated for different thresholds to understand the accuracy of the test. Here, the EORTC QLQ-C30 scale scores form our numerical predictors and our binary outcomes are supportive care need on the specific anchors chosen (need vs no need). Sensitivity here indicates the proportion of individuals that score worse than the cut-off that truly have supportive care need on the anchor (score > 2). Specificity indicates the proportion of individuals that score better than the cut-off that truly do not have supportive care need on the anchor (score ≤ 2).

We then calculated the AUC to determine the EORTC QLQ-C30 scale scores’ ability to discriminate between patients with need and those with no need on the selected anchors [21]. There is no agreed definition for an adequate AUC score, though evidence suggests that values below 0.70 indicate poor discrimination, values between 0.70 and 0.80 indicate acceptable discrimination and values above 0.80 indicate excellent discrimination [22]. If the AUC was below 0.70, cut-offs with sensitivity and specificity were not calculated. This AUC indicates the EORTC QLQ-C30 score would not adequately identify patients with and without need on the chosen anchor and should not be used as a screening tool.

Where the AUC exceeded 0.70, we calculated the cut-offs with associated sensitivities and specificities. We selected the optimal cut-off by maximising Youden’s J statistic (the sum of sensitivity and specificity minus one). If the statistics for two adjacent thresholds differed by less than 0.05 we selected the threshold with the higher sensitivity following the methods described by Giesinger et al. [14]. Where the sensitivity for the cut-off these parameters indicated was below 0.70, we chose the closest threshold with a sensitivity above this value where possible.

### Sensitivity analyses

#### Invariance by treatment status

For EORTC QLQ-C30 scores with agreed anchors, we conducted sensitivity analyses to explore variability in diagnostic accuracy and optimal cut-off scores by treatment status (on treatment vs. on follow-up). To determine the diagnostic accuracy, we calculated the AUC for each EORTC QLQ-C30 scale separately for patients on treatment and on follow-up. To examine variability in optimal cut-off scores, we used a multivariate logistic regression model for each chosen pair of SCNS-LF59 anchors and EORTC QLQ-C30 domains. In each model, the SCNS-LF59 binary anchor was included as the dependent variable. The EORTC QLQ-C30 domain score and treatment status were included as independent variables. If treatment status was significantly associated with the anchor (*p* < 0.01), this indicated the optimal cut-off score was different between groups. In these cases, we calculated the cut-off score separately for patients on treatment and on follow-up and chose the optimal scores based on the criteria mentioned above. We also calculated the sensitivity and specificity of the new cu—off scores and compared them to the total sample.

#### Invariance by anchor selection method

Previous analyses selected anchors based on the highest AUC rather than expert opinion. To explore the impact of including multidisciplinary experts in the selection of anchors, we repeated the analysis using the anchors with the highest AUC and compared the findings where the anchors differed.

## Results

### Sample characteristics

A total of 347 YAs completed the survey of 1683 (20.6%) YAs invited between May 2018 and October 2019. Three hundred and thirteen participants had complete data and were included in analysis. On average, YAs were 33.3 years old (SD 4.2) at diagnosis and 2.8 years from diagnosis (SD 1.6) (Table 1). The majority of participants were female (*N* = 216; 69.0%), of white descent (*N* = 268; 85.6%) and university educated (*N* = 202; 64.5%). Participants most commonly had breast cancer (*N* = 100; 31.9%), were on follow-up (*N* = 238; 76.0%) and were treated with curative intent (*N* = 244; 76.7%).

**Table 1:** Summary of demographic and clinical participant details

Characteristic (*N* = 313)	Mean [SD]		Range
Mean age at diagnosis in years	33.3 [4.2]		25–39
Mean current age in years	36.1 [4.5]		26–45
Years from diagnosis	2.8 [1.6]		0–7

	Frequency	Percentage
Gender		
Female	216	69.0%
Male	97	31.0%
Ethnicity		
White	268	85.6%
Asian/Asian British	26	8.3%
Mixed/Multiple ethnic groups	12	3.8%
Black/African/Caribbean/Black British	3	1.0%
Other ethnic group	4	1.3%
Educational attainment		
University	202	64.5%
College/diploma	59	18.8%
Secondary school	30	9.6%
Vocational qualification	16	5.1%
Primary school	2	0.6%
Other	4	1.3%
Diagnosis		
Breast cancer	100	31.9%
Testicular cancer	47	15.0%
Gynaecological cancers	44	14.1%
Haematological malignancies	36	11.5%
Sarcomas	26	8.3%
Head & neck cancers	23	7.4%
Gastrointestinal cancers	14	4.5%
Melanoma	11	3.5%
Other	12	3.8%
Treatments received (non-exclusive)		
Surgery	247	78.9%
Chemotherapy	182	58.1%
Radiotherapy	141	45.0%
Hormone therapy	64	20.4%
Clinical trial therapy	34	10.9%
Complementary therapy	28	9.0%
Targeted therapy	28	9.0%
Immunotherapy	18	5.6%
Active surveillance	13	4.2%
Stem cell transplant	7	2.2%
Other	29	9.3%
Current treatment status		
On follow-up	238	76.0%
On treatment	75	24.0%
Treatment intent		
Curative	244	76.7%
Palliative	46	14.7%
Unknown	25	8.0%
Missing	2	0.6%

*SD* standard deviation

### Cut-offs for supportive care need

Six experts chose to take part in anchor selection including two YA patients from the United Kingdom, two clinical psychologists from Austria and two quality of life researchers from Austria and the Netherlands. Experts agreed on anchors for ten of the 15 of the EORTC QLQ-C30 scales after two rounds of ratings (Table 2). All potential anchors were excluded for Cognitive Functioning, Dyspnoea, Constipation, Appetite Loss and Diarrhoea as the SCNS-LF59 lacked items with similar content. The AUC for each agreed anchor ranged from 0.78 to 0.87 (Table 2). The highest AUCs were observed for Nausea and Vomiting (0.867) and Pain (0.865) and the lowest AUCs were observed for Financial Difficulties (0.776) and Global Quality of Life (0.781).

**Table 2:** Results of the receiver operating characteristic (ROC) analysis for scales with agreed anchors and cut-off scores

EORTC QLQ-C30 scale	SCNS-LF59 anchor	Cut-Off Score	AUC (95% CI)	Sensitivity (95% CI)	Specificity (95% CI)
Global quality of life	Feeling unwell a lot of the time + Not being able to do the things you used to do	71	0.78 (0.71–0.85)	0.78 (0.68–0.86)	0.69 (0.64–0.75)
Physical functioning	Physical and daily living needs	97	0.79 (0.73–0.85)	0.80 (0.71–0.89)	0.61 (0.54–0.67)
Role functioning	Not being able to do the things you used to do	92	0.84 (0.78–0.89)	0.82 (0.74–0.91)	0.74 (0.67–0.79)
Emotional functioning	Psychological needs	71	0.79 (0.74–0.84)	0.71 (0.64–0.79)	0.70 (0.64–0.77)
Social functioning	Changes to usual routine and lifestyle + Changes in other peoples attitudes towards you	92	0.81 (0.75–0.86)	0.82 (0.75–0.90)	0.70 (0.64–0.77)
Fatigue	Lack of energy/tiredness	28	0.84 (0.79–0.88)	0.78 (0.69–0.85)	0.79 (0.73–0.85)
Nausea and vomiting	Nausea/vomiting	8	0.87 (0.79–0.94)	0.83 (0.69–0.97)	0.88 (0.84–0.92)
Pain	Pain	8	0.87 (0.82–0.91)	0.88 (0.79–0.95)	0.74 (0.69–0.80)
Insomnia	Not sleeping well	17	0.83 (0.78–0.88)	0.87 (0.80–0.93)	0.66 (0.60–0.72)
Financial difficulties	Concerns about your financial situation	17	0.78 (0.72–0.83)	0.64 (0.54–0.74)	0.88 (0.84–0.92)

*EORTC QLQ-C30* European Organisation for Research and Treatment of Cancer Quality of Life Questionnaire Core 30, *SCNS-LF59* supportive care needs survey long form 59, *AUC* area under the ROC curve, *CI* confidence interval

Cut-offs for the functioning scales and Global Quality of Life, where higher scores indicate better functioning, ranged from 71 for Global Quality of Life and Emotional Functioning to 97 for Physical Functioning (Table 2). Cut-offs for the symptom scales, where higher scores indicate more problems, ranged from 8 for Nausea and Vomiting and Pain to 17 for Insomnia and Financial Difficulties. Sensitivity ranged from 0.64 for Financial Difficulties to 0.88 for Pain (Table 2). Specificity ranged from 0.61 for Physical Functioning to 0.88 for Nausea and Vomiting and Financial Difficulties (Table 2).

The proportion of patients with need on the chosen anchors for each scale ranged from 9.3% on Nausea and Vomiting to 42.2% on Emotional Functioning (Table 3). The largest difference in EORTC QLQ-C30 mean score between patients with and without need in effect size was found for Nausea and Vomiting (Cohen’s *d* =  − 1.74) and Pain (Cohen’s *d* =  − 1.44) and the smallest difference was found for Emotional Functioning (Cohen’s *d* = 1.00) and Global Quality of Life (Cohen’s *d* = 1.11).

**Table 3:** Number of patients with and without supportive care need on each SCNS-LF59 anchor and corresponding EORTC QLQ-C30 scale score

EORTC QLQ-C30 Scale	SCNS-LF59 anchor	Respondents with *no need* on SCNS-LF59 anchor		Respondents with *need* on SCNS-LF59 anchor		Difference in mean score	Effect size^a^
		Number (%)	Mean EORTC QLQ-C30 scale score (SD)	Number (%)	Mean EORTC QLQ-C30 scale score (SD)		
Global quality of life^b^	Feeling unwell a lot of the time + Not being able to do the things you used to do	240 (76.9)	78.3 (16.3)	72 (23.1)	55.2 (24.5)	23.1	1.11
Physical functioning^b^	Physical and daily living needs	226 (72.2)	95.1 (7.8)	87 (27.8)	75.6 (23.1)	19.5	1.20
Role functioning^b^	Not being able to do the things you used to do	227 (72.8)	91.6 (17.7)	85 (27.2)	53.9 (32.7)	37.6	1.33
Emotional functioning^b^	Psychological needs	181 (57.8)	80.8 (18.9)	132 (42.2)	54.9 (26.8)	25.8	1.00
Social functioning^b^	Changes to usual routine and lifestyle + changes in other peoples attitudes towards you	217 (69.3)	89.6 (19.2)	96 (30.7)	58.2 (30.8)	31.4	1.14
Fatigue^c^	Lack of energy/tiredness	192 (61.5)	17.0 (20.7)	120 (38.5)	49.3 (27.8)	− 32.3	− 1.14
Nausea and vomiting^c^	Nausea/vomiting	283 (90.7)	3.1 (10.6)	29 (9.3)	28.2 (24.0)	− 25.0	− 1.74
Pain^c^	Pain	239 (76.6)	7.5 (15.2)	73 (23.3)	42.9 (29.8)	− 35.5	− 1.44
Insomnia^c^	Not sleeping well	222 (72.1)	15.2 (24.5)	86 (27.9)	55.8 (32.9)	− 40.6	− 1.25
Financial difficulties^c^	Concerns about your financial situation	221 (70.6)	5.4 (16.5)	92 (29.4)	40.9 (37.3)	− 35.5	− 1.21

*EORTC QLQ-C30* European Organisation for Research and Treatment of Cancer Quality of Life Questionnaire Core 30, *SCNS-LF59* supportive care needs survey long form 59, *SD* standard deviation

^a^Cohen’s *d* based on pooled standard deviation

^b^Higher scores indicate better functioning or quality of life

^c^Better scores indicate worse symptom burden

### Sensitivity analyses

#### Invariance by treatment status

We examined the diagnostic accuracy and optimal cut-off scores by treatment status for the ten EORTC QLQ-C30 scales with agreed anchors. All 20 AUCs were above 0.70 indicating acceptable discrimination (Table 4). AUCs for both groups were above 0.80 indicating excellent discrimination for Role Functioning, Nausea and Vomiting, and Pain. AUCs were also above 0.80 for Emotional Functioning and Insomnia for patients on treatment and Fatigue for patients on follow-up.

**Table 4:** Results of the sensitivity analyses for scales with optimal cut-off scores that vary by treatment status

EORTC QLQ-C30 Scale	SCNS-LF59 Anchor	Group	Cut-Off Score	AUC (95% CI)	Sensitivity (95% CI)	Specificity (95% CI)
Global quality of life	Feeling unwell a lot of the time + Not being able to do the things you used to do	Total	71	0.78 (0.71–0.85)	0.78 (0.68–0.86)	0.69 (0.64–0.75)
		On TX	63	0.78 (0.67–0.89)	0.73 (0.58–0.88)	0.83 (0.71–0.93)
		On FU	71	0.75 (0.66–0.85)	0.74 (0.59–0.87)	0.72 (0.65–0.78)
Physical functioning	Physical and daily living needs	Total	97	0.79 (0.73–0.85)	0.80 (0.71–0.89)	0.61 (0.54–0.67)
		On TX	90	0.75 (0.64–0.86)	0.74 (0.61–0.87)	0.59 (0.43–0.76)
		On FU	97	0.77 (0.69–0.85)	0.76 (0.63–0.88)	0.64 (0.57–0.71)
Emotional functioning	Psychological needs	Total	71	0.79 (0.74–0.84)	0.71 (0.64–0.79)	0.70 (0.64–0.77)
		On TX	71	0.83 (0.73–0.93)	0.74 (0.63–0.85)	0.76 (0.57–0.90)
		On FU	79	0.76 (0.70–0.83)	0.79 (0.71–0.88)	0.55 (0.48–0.63)
Financial difficulties	Concerns about your financial situation	Total	17	0.78 (0.72–0.83)	0.64 (0.54–0.74)	0.88 (0.84–0.92)
		On TX	17	0.79 (0.70–0.88)	0.70 (0.55–0.85)	0.80 (0.66–0.91)
		On FU	17	0.75 (0.68–0.83)	0.60 (0.46–0.73)	0.90 (0.85–0.94)

*EORTC QLQ-C30* European Organisation for Research and Treatment of Cancer Quality of Life Questionnaire Core 30, *SCNS-LF59* supportive care needs survey long form 59, *AUC* area under the ROC curve, *CI* confidence interval, *TX* treatment, *FU* follow-up

Cut-off scores differed by group for four domains (*p* < 0.01). The optimal cut-off scores for patients on treatment were lower than those for the total sample on Global Quality of Life and Physical Functioning (Table 4). For the on treatment group, the optimal cut-off score on Financial Difficulties was the same but sensitivity reached the acceptable threshold. For Emotional Functioning, the optimal cut-off score for patients on follow-up was higher than for the total sample and had better sensitivity.

#### Invariance by anchor selection method

The anchors for four scales (Global Quality of Life, Physical Functioning, Emotional Functioning and Social Functioning) differed when chosen according to the highest AUC rather than expert opinion (Table 5). The AUC for the composite anchor chosen by experts for ‘Global Quality of Life’ was higher than the single SCNS-LF59 item with the highest AUC (0.781 vs 0.761, respectively). The expert chosen anchor also had slightly higher sensitivity (0.78 vs 0.74, respectively), though the cut-off was the same. The AUCs of all other anchors selected by experts were lower than those chosen according to AUC.

**Table 5:** Comparison of anchors selected using expert rationale compared to selection according to highest area under the receiver operator characteristic (ROC) curve

EORTC QLQ-C30 scale	Method	SCNS-LF59 anchor	Cut-Off Score	AUC (95% CI)	Sensitivity (95% CI)	Specificity (95% CI)
Global quality of life	Expert	Feeling unwell a lot of the time + Not being able to do the things you used to do	71	0.78 (0.71–0.85)	0.78 (0.68–0.86)	0.69 (0.64–0.75)
	AUC	Changes to usual routine and lifestyle	71	0.76 (0.70–0.82)	0.74 (0.66–0.83)	0.72 (0.72–0.78)
Physical functioning	Expert	Physical and daily living needs	97	0.79 (0.73–0.85)	0.80 (0.71–0.89)	0.61 (0.54–0.67)
	AUC	Work around the home	90	0.82 (0.74–0.89)	0.79 (0.79–0.98)	0.75 (0.49–0.55)
Emotional functioning	Expert	Psychological needs	71	0.79 (0.74–0.84)	0.71 (0.64–0.79)	0.70 (0.64–0.77)
	AUC	Feelings of sadness	79	0.85 (0.81–0.90)	0.88 (0.82–0.93)	0.63 (0.55–0.70)
Social functioning	Expert	Changes to usual routine and lifestyle + Changes in other peoples attitudes towards you	92	0.81 (0.75–0.86)	0.82 (0.75–0.90)	0.70 (0.64–0.77)
	AUC	Changes to usual routine and lifestyle	92	0.84 (0.79–0.88)	0.86 (0.79–0.93)	0.71 (0.65–0.77)

*EORTC QLQ-C30* European Organisation for Research and Treatment of Cancer Quality of Life Questionnaire Core 30, *SCNS-LF59* supportive care needs survey long form 59, *AUC* area under the ROC curve, *CI* confidence interval

The cut-offs for two scales (Physical Functioning and Emotional Functioning) differed when chosen according to the highest AUC rather than expert opinion. The cut-off for Physical Functioning was less severe using the anchor chosen by the experts compared to the anchor chosen according to highest AUC (97 vs 90, respectively). However, these cut-offs had similar sensitivity (0.80 vs 0.79, respectively). The cut-off for Emotional Functioning was more severe when using the anchor chosen by experts compared to the anchor chosen according to highest AUC (71 vs 79, respectively). Sensitivity for the anchor chosen by experts was lower than for Emotional Functioning than the anchor chosen according to highest AUC (0.71 vs 0.88, respectively).

## Discussion

We identified cut-offs for ten of the 15 EORTC QLQ-C30 scales with adequate to exceptional ability to discriminate between YA cancer patients with and without need for support. Most cut-offs identified here have good sensitivity, indicating that the majority of patients who score worse than the threshold will have a true need for support. This is the first study to establish cut-offs for a major PRO measure for YAs with cancer.

The exception is ‘Financial Difficulties’ which did not meet the requirement for sensitivity of at least 0.70. Using the threshold of 17 on ‘Financial Difficulties’ will miss about 35% of YAs that need support. This was surprising given the similarity in content between the EORTC QLQ-C30 scale and the SCNS-LF59 anchor. This may reflect inconsistencies in patient’s perception of the healthcare system’s ability to provide support for financial concerns. If a respondent felt the healthcare team would be unable to provide support, they may not report that they have ‘need’ even if they have financial issues. Financial toxicity is high amongst YAs compared to older adults and about a third of patients had need for financial concerns in this study [23]. Future research should prioritise developing appropriate methods to identify and address financial toxicity amongst YAs.

The sensitivity analyses showed that four of the ten optimal cut-off scores differed between YAs on treatment and YAs on follow-up. Given the relatively small number of patients on treatment in our sample, this work should be considered valid for patients on follow-up and replicated in YAs on treatment to determine the most appropriate scores to use for this group. Whilst the optimal Emotional Functioning cut-off score for patients on follow-up was found to be higher than the score for the total sample, it may be preferable to use the lower score in a screening setting to ensure all patients with psychological need are captured.

Excluding the cut-off for Financial Difficulties given its poor sensitivity, we were able to identify three more cut-offs compared to Snyder et al.’s previous analysis [11, 12]. These new cut-offs for Social Functioning, Nausea and Vomiting and Insomnia were identified using items in the SCNS-LF59 not previously included in the SCNS short form.

The cut-offs identified here were similar to those identified by Snyder et al. except for emotional and role functioning, where we identified more lower or worse scores as the cut-offs. This may reflect differences between adults and YAs. YAs may have more informal emotional support from friends and family than older adults which may translate into less perceived need for formal support from the healthcare team resulting in more lower cut-offs. Alternatively, YAs may not report the need for support if they think no relevant services in the healthcare system can address the issue. This may explain the wore score for role functioning which was anchored to ‘Not being able to do the things you used to do’ in our study compared to ‘Work around the home’ in prior studies.

In contrast, the cut-offs identified by Giesinger et al. were similar or lower. This likely reflects the different conceptualisation of the cut-offs. A symptom or functional problem may need to be more severe to be worrying or life limiting than to be interested in support. This may also reflect the fact that YAs may have higher expectations towards their level of functioning compared to older adults. In addition, our sample largely comprised survivors no longer on treatment who may again have higher expectations for symptoms and functional status compared to patients on treatment, and thus report supportive care needs at less severe scores.

These cut-offs can facilitate clinical interpretation for use of the EORTC QLQ-C30 in routine care by indicating which scores require clinical attention. For example, the scores can be integrated into the medical record by presenting clinicians with graphs highlighting the scores that indicate supportive care is needed [24]. The involvement of YA patients, clinical psychologists and health researchers ensured matching SCNS-LF59 anchors to EORTC QLQ-C30 scales was based on theory and experience rather than statistics alone. It was interesting to find that the composite anchor for Global Quality of Life had a higher AUC than any single item alone, supporting the selection of anchors based on expert rationale rather than statistical methods. Although including patient, clinician and researcher experts in the selection of anchors results in similar cut-offs compared to relying on the selection of anchors using statistical methods, namely maximising the AUC.

In Physical Functioning, the cut-off was less severe though with similar sensitivity when the anchor was selected by experts. Particularly as this is a young population, any reduction in physical function may be more likely to be unusual and more damaging to quality of life and therefore require more clinical attention. For example, the most vigorous item on the Physical Functioning scale is taking a long walk. Older patients may have such limitations for other reasons whilst the inability to perform such basic activities may be more concerning for a young person. Therefore, the cut-off score of 97 defined by the expert chosen anchor would be recommended. In contrast, the Emotional Functioning cut-off was more severe when the anchor was chosen by experts. This may reflect that emotional function is more than feeling sad (the anchor with the highest AUC) and that sadness alone when experiencing a cancer diagnosis does not necessitate support. However, the AUC and sensitivity for the expert chosen anchor is much lower than the AUC-defined anchor. As these cut-off scores would be used in a screening setting, we would recommend taking the cautious approach and using the less severe cut-off score of 79.

These cut-offs are beneficial in that they are simple for clinicians to use to identify supportive care need using the EORTC QLQ-C30 in routine care. However, screening tools always have a trade-off between sensitivity and specificity. This means the EORTC QLQ-C30 will falsely indicate some patients have need and falsely indicate others do not have need. Here we favoured sensitivity over specificity by setting a minimum requirement of 0.70 sensitivity. This means patients with need are unlikely to be missed. Favouring sensitivity does, however, increase the number of false positives. In this context, this seems favourable as the result of a positive is a clinical discussion rather than invasive investigation, however, this could lead to alert fatigue for the clinician. Trials that have used similar approaches have not found a significant increase in workload, however, alert fatigue would need to be explored in an empirical evaluation of the cut-offs [3].

### Limitations

As a few of the scales in the EORTC QLQ-C30 are made up of only one or two items, there is limited precision in the measurement of the concept and therefore, the potential thresholds. This contributes to large differences between sensitivity and specificity, meaning that to achieve adequate sensitivity, there will be lower specificity and potentially a high number of false positives. False positives could lead to ‘burnout’ and rejection of the use of such a screening method in clinical practice. Using a quality of life instrument with higher precision may improve the sensitivity and specificity of the cut-offs as seen in the development of cut-offs for the computer adaptive test version of the EORTC QLQ-C30 in further work by Giesinger et al. [9].

Further work to compare and validate cut-offs to determine the most appropriate instruments and thresholds is necessary given the potential impact of their use in practice. Whilst the SCNS-LF59 is well validated in cancer populations, it may miss some supportive care needs specific to YAs such as support returning to work, managing childcare or physical activity advice. In addition, the SCNS-LF59 is a self-report of need, which may be influenced by other factors such as knowledge of the availability of support or beliefs about the effectiveness of services. However, we view the use of a self-report measure of need as a strength as it is face valid, clinically relevant and values patient views. This ensures we take a patient-centred approach to supportive care provision.

The survey had a low response rate which may limit the generalisability of the study. In particular, the survey favoured female YAs with a high level of education, no longer on treatment and those with breast cancer. YAs included in this sample may have been higher functioning than the broader population of YAs with cancer leading to higher cut-off scores than necessary in general practice. The high proportion of females means the findings may not generalise well to males, although previous studies have found cut-off scores do not vary by gender [9, 14]. Future research should aim to validate these findings.

## Conclusions

We identified nine appropriate cut-offs for supportive care needs on the EORTC QLQ-C30 for YAs with cancer in follow-up. This is the first study to establish cut-offs for a major PRO measure for YAs with cancer. The use of these thresholds will facilitate the measurement of quality of life routinely in cancer care to help identify those with need. Further investigation to empirically compare these cut-offs to others is necessary to select the most appropriate metrics depending on the purpose and population. Additional research is also needed to look at cut-off scores for clinically significant changes in longitudinal measurement in clinical care.

## Data Availability

Data not available.

## References

[1] Lohr KN, Zebrack BJ (2009). Using patient-reported outcomes in clinical practice: Challenges and opportunities. Quality of Life Research.

[2] Collaboration TC. Cochrane Handbook for Systematic Reviews of Interventions: Cochrane Book Series. 5.1.0. Higgins J, Green S, editors. Wiley, New York, 2008.

[3] Ishaque S, Karnon J, Chen G, Nair R, Salter AB (2019). A systematic review of randomised controlled trials evaluating the use of patient-reported outcome measures (PROMs). Quality of Life Research.

[4] Basch E, Deal AM, Dueck AC, Scher HI, Kris MG, Hudis C (2017). Overall survival results of a trial assessing patient-reported outcomes for symptom monitoring during routine cancer treatment. JAMA - J Am Med Assoc..

[5] Maguire R, McCann L, Kotronoulas G, Kearney N, Ream E, Armes J (2021). Real time remote symptom monitoring during chemotherapy for cancer: European multicentre randomised controlled trial (eSMART). BMJ.

[6] Absolom K, Warrington L, Hudson E, Hewison J, Morris C, Holch P (2021). Phase III randomized controlled trial of eRAPID: EHealth intervention during chemotherapy. Journal of Clinical Oncology.

[7] Gibbons C, Porter I, Gonçalves-Bradley DC, Stoilov S, Ricci-Cabello I, Tsangaris E (2021). Routine provision of feedback from patient-reported outcome measurements to healthcare providers and patients in clinical practice. Cochrane Database of Systematic Reviews.

[8] Nguyen H, Butow P, Dhillon H, Sundaresan P (2021). A review of the barriers to using patient-reported outcomes (PROs) and patient-reported outcome measures (PROMs) in routine cancer care. Journal of Medical Radiation Sciences.

[9] Giesinger JM, Loth FLC, Aaronson NK, Arraras JI, Caocci G, Efficace F (2020). Thresholds for clinical importance were defined for the European Organisation for Research and Treatment of Cancer Computer Adaptive Testing Core—an adaptive measure of core quality of life domains in oncology clinical practice and research. Journal of Clinical Epidemiology.

[10] Giesinger JM, Aaronson NK, Arraras JI, Efficace F, Groenvold M, Kieffer JM (2018). A cross-cultural convergent parallel mixed methods study of what makes a cancer-related symptom or functional health problem clinically important. Psycho-Oncology.

[11] Snyder CF, Blackford AL, Brahmer JR, Carducci MA, Pili R, Stearns V (2010). Needs assessments can identify scores on HRQOL questionnaires that represent problems for patients: An illustration with the Supportive Care Needs Survey and the QLQC30. Quality of Life Research.

[12] Snyder CF, Blackford AL, Okuyama T, Akechi T, Yamashita H, Toyama T (2013). Using the EORTC-QLQ-C30 in clinical practice for patient management: Identifying scores requiring a clinician’s attention. Quality of Life Research.

[13] Boyes A, Girgis A, Lecathelinais C (2009). Brief assessment of adult cancer patients’ perceived needs: Development and validation of the 34-item Supportive Care Needs Survey (SCNS-SF34). Journal of Evaluation in Clinical Practice.

[14] Giesinger JM, Loth FLC, Aaronson NK, Arraras JI, Caocci G, Efficace F (2020). Thresholds for clinical importance were established to improve interpretation of the EORTC QLQ-C30 in clinical practice and research. Journal of Clinical Epidemiology.

[15] Van De Poll-Franse LV, Horevoorts N, Van EM, Denollet J, Roukema JA, Aaronson NK (2011). The patient reported outcomes following initial treatment and long term evaluation of survivorship registry: Scope, rationale and design of an infrastructure for the study of physical and psychosocial outcomes in cancer survivorship cohorts. European Journal of Cancer.

[16] Clinton-McHarg T, Carey M, Sanson-Fisher R, D’Este C, Shakeshaft A (2012). Preliminary development and psychometric evaluation of an unmet needs measure for adolescents and young adults with cancer: The Cancer Needs Questionnaire - Young People (CNQ-YP). Health and Quality of Life Outcomes.

[17] Bonevski B, Sanson-Fisher R, Girgis A, Burton L, Cook P, Boyes A (2000). Evaluation of an instrument to assess the needs of patients with cancer. Cancer.

[18] Aaronson NK, Ahmedzai S, Bergman B, Bullinger M, Cull A, Duez NJ (1993). The European Organization for Research and Treatment of Cancer QLQ-C30: A quality-of-life instrument for use in international clinical trials in oncology. JNCI Journal of National Cancer Institute.

[19] Fayers PM, Aaronson NK, Bjordal K, Grønvold M, Curran D, Bottomley A (2001). EORTC QLQ-C30 Scoring Manual.

[20] Zou KH, O’Malley AJ, Mauri L (2007). Receiver-operating characteristic analysis for evaluating diagnostic tests and predictive models. Circulation.

[21] Mandrekar JN (2010). Receiver operating characteristic curve in diagnostic test assessment. Journal of Thoracic Oncology.

[22] Hosmer DW, Lemeshow S (2000). Applied logistic regression.

[23] Salsman JM, Kircher SM (2021). Financial hardship in adolescent and young adult oncology: The need for multidimensional and multilevel approaches. JCO Oncology Practice.

[24] Snyder C, Smith K, Holzner B, Rivera Y, Bantug E, Brundage M (2019). Making a picture worth a thousand numbers: Recommendations for graphically displaying patient-reported outcomes data. Quality of Life Research.

